# Protonation of Pt(IV) Anticancer Complexes Assayed by Vibrational Ion Spectroscopy

**DOI:** 10.1002/cplu.202400754

**Published:** 2025-04-03

**Authors:** Davide Corinti, Elisabetta Gabano, Barbara Chiavarino, Maria Elisa Crestoni, Domenico Osella, Simonetta Fornarini

**Affiliations:** ^1^ Dipartimento di Chimica e Tecnologie del Farmaco Università di Roma “La Sapienza” P. le A. Moro 5 I-00185 Roma Italy; ^2^ Dipartimento per lo Sviluppo Sostenibile e la Transizione Ecologica Università del Piemonte Orientale Piazza S. Eusebio 5 13100 Vercelli Italy; ^3^ Dipartimento di Scienze e Innovazione Tecnologica Università del Piemonte Orientale Viale T. Michel 11 15121 Alessandria Italy

**Keywords:** Bioinorganic chemistry, Mass spectrometry, Density functional calculations, Anticancer agents

## Abstract

Platinum(IV) complexes are being studied as potential alternatives to traditional platinum(II)‐based chemotherapy drugs. They promise reduced side effects and potential for oral administration. In fact, a preliminary reduction in the cellular medium is recognized as a crucial step for activation. However, a deeper understanding of the protonation sites and substitution behavior of Pt(IV) complexes is needed, considering that ligand hydrolysis may compete with reduction‐mediated activation, particularly in acidic environments such as the stomach. In this study, we investigated protonated Pt(IV) complexes with equatorial ligands common to widely used Pt(II) drugs containing square planar geometry, such as cisplatin and carboplatin. The additional axial substituents in the octahedral coordination sphere of Pt(IV) include different combinations of hydroxido and acetato ligands. Mass spectrometry‐based methods, including collision‐induced dissociation (CID) and infrared multiple photon dissociation (IRMPD) spectroscopy, supported by density functional theory (DFT) calculations, were employed. Structural characterization revealed that protonation preferences are influenced by the type and position of the ligands. Notably, protonation is generally favored on the carboxylato ligands; however, the carboplatin‐derived complex exhibited a mixed population of protomers, highlighting the significance of both axial and equatorial ligand configurations in shaping the prototropic equilibria happening in solution.

## Graphical abstract

Protonated Pt(IV) complexes with various equatorial and axial ligands were characterized by IR ion spectroscopy and DFT. Protonation typically occurs at carboxylato ligands, eventually increasing their substitution propensity. However, the carboplatin‐derived complex showed mixed protomer populations, highlighting the significance of ligand selection to mitigate undesired reactivity.

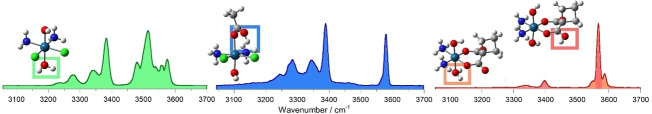

## Introduction

**Scheme 1:**
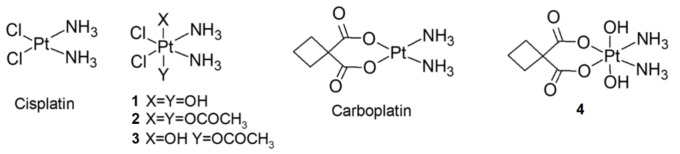
Schematic representation of the assayed Pt(IV) complexes. The structures of cisplatin and carboplatin are also reported.

Platinum is the exogenous element that has found the most prominent role in therapeutic applications. Platinum complexes are routinely used to treat various solid tumors, including lung, testicular, and ovarian cancers.[^1^, ^2^, ^3^] Currently, all FDA‐approved platinum‐containing drugs are square‐planar Pt(II) compounds with the general formula *cis*‐[PtX₂L₂], where X represents labile ligands prone to substitution reactions with nucleophiles, and L refers to inert ligands, typically nitrogen‐based nucleophiles such as ammonia or substituted amines.^[4]^ Cisplatin (Scheme 1) is the simplest and most widely studied member of this family of platinating agents. It contains two chlorides and two ammonia groups in the coordination sphere of the metal. The chlorido ligands are readily substituted by water in cellular environments, giving rise to the diaqua complex of cisplatin (*cis*‐[Pt(NH_3_)_2_(OH_2_)_2_]^2+^), a positively charged species that is considered the active form of the drug.[^5^, ^6^] This species interacts with the nucleobases of DNA producing inhibition of transcription and eventually cell‐death.[^5^, ^7^, ^8^] Despite their clinical utility, all approved platinum drugs exhibit significant adverse effects and are administered via slow intravenous infusion. This requirement reduces patient compliance and impacts quality of life. In contrast, platinum(IV) complexes offer features which enable their oral administration, thus overcoming some limitations associated with cisplatin treatment.[^9^, ^10^, ^11^, ^12^, ^13^] Pt(IV) complexes exhibit octahedral geometry and are more inert to ligand substitution. Thus, a preliminary reduction of the metal, involving the loss of the axial ligands, is required to activate the complex for its interaction with DNA.[^10^, ^14^, ^15^, ^16^] The reduction and activation mechanisms of Pt(IV) antineoplastic complexes have been extensively studied, including computational analyses,[^17^, ^18^, ^19^] and experimental determinations of cellular reductants involved in these processes.[^20^, ^21^, ^22^, ^23^, ^24^, ^25^] However, a universal consensus on the inert nature of Pt(IV) ligands is lacking.[^26^, ^27^, ^28^, ^29^, ^30^, ^31^, ^32^, ^33^, ^34^, ^35^, ^36^, ^37^] This is particularly relevant in acidic environments, such as the stomach, where ligand hydrolysis could occur, potentially altering the activation pathway.^[27]^

In order to gain knowledge on ligand susceptibility to substitution in acidic environments, we have explored the protonation sites of Pt(IV) complexes exhibiting diverse equatorial and axial ligands. Assayed species include **1** (*cis,cis,trans*‐[PtCl_2_(NH_3_)_2_(OH)_2_]), **2** (*cis,cis,trans*‐[PtCl_2_(NH_3_)_2_(OCOCH_3_)_2_]), **3** (*cis,cis,trans*‐[PtCl_2_(NH_3_)_2_(OCOCH_3_)(OH)]) and **4** (*cis,cis,trans*‐[Pt(C_6_H_6_O_4_)(NH_3_)_2_(OH)_2_]) (scheme 1). The first three complexes have a common equatorial scaffold and differ just for the axial ligands. In particular, the complexes **1**, **2** and **3** have a cisplatin‐like structure with two ammonia and two chlorides in *cis* configuration as equatorial ligands, while in the axial positions **1** present two hydroxo groups, **2** two acetates, and **3** a mixed configuration with one acetato and one hydroxido ligand. In contrast, **4** replaces the equatorial chlorides with a 1,1‐cyclobutanedicarboxylato chelating ligand, the labile component of the antineoplastic drug carboplatin (scheme 1).

To structurally characterize the protonated complexes and generate information on the ligands that are more easily released in the gas phase, mass spectrometry‐based methods have been employed. This combined approach, consisting of collision‐induced dissociation (CID) experiments and IR multiple photon dissociation (IRMPD) spectroscopy backed by calculations at the DFT level,[^38^, ^39^, ^40^, ^41^, ^42^] has proven effective in obtaining structural information on different types of Pt complexes, relevant to both medicinal chemistry,[^25^, ^43^, ^44^, ^45^, ^46^, ^47^] and catalysis.[^48^, ^49^, ^50^] Here, the protonation sites in the presence of diverse ligands were determined. The gathered information is intended to assist in the design of Pt(IV) complexes with an awareness of the potential for substitution in certain portions of the complex.

## Experimental Section

### Materials and MS Analysis

The investigated complexes, namely (*OC*‐6‐33)‐diamminedichloridodihydroxidoplatinum(IV) (oxoplatin, **1**, *cis,cis,trans*‐[PtCl_2_(NH_3_)_2_(OH)_2_]), (*OC*‐6‐33)‐diacetatodiamminedichloridoplatinum(IV) (**2**, *cis,cis,trans*‐[PtCl_2_(NH_3_)_2_(OCOCH_3_)_2_]), (OC‐6‐44)‐acetatodiamminedichloridohydroxidoplatinum(IV) (**3**, *cis,cis,trans*‐[PtCl_2_(NH_3_)_2_(OCOCH_3_)(OH)]) and (*OC*‐6‐33)‐diammine(cyclobutane‐1,1‐dicarboxylato)dihydroxidoplatinum(IV) (**4**, *cis,cis,trans*‐[Pt(C_6_H_6_O_4_)(NH_3_)_2_(OH)_2_]) have been prepared as already described in literature.[^51^, ^52^, ^53^, ^54^, ^55^]

Samples were prepared by diluting the complexes in a water/methanol 50 : 50 v/v solution to a final concentration of 10^−5^ M. Formic acid was added to the solutions to increase protonation reaching a final concentration of 0.1 % v/v.

Mass spectrometry analyses were conducted using a Paul ion trap (Esquire 6000, Bruker) equipped with an electrospray ionization (ESI) source. Solutions were directly infused in the ESI source at a flow rate of 180 μL h^−1^. Parameters employed for the analyses were: capillary voltage at −3.8 kV, drying gas (N_2_) fluxed at 7 L/min at temperature of 300 °C, capillary exit at 60 V and skimmer voltage at 45 V. CID experiments have been performed on mass‐selected ions using an activation amplitude of 0.35 V and an activation time of 50 ms. Helium was employed as collision gas at a nominal pressure of 1.5×10^−6^ mbar.

Additional CID experiments were carried out using a linear ion trap mass spectrometer (LTQ XL, Thermo Scientific) coupled with an ESI source. Solutions were infused at a flow rate of 360 μL h^−1^. ESI parameters were set as follows: temperature at 250 °C, spray voltage at 5 kV, sheath gas flow rate at 10 arb. u., aux gas flow rate at 2 arb. u., capillary voltage at 20 V, and tube lens voltage at 50 V.

### IRMPD Spectroscopy

IR multiple photon dissociation (IRMPD) spectra have been obtained in the X−H (X= C, N, O) stretching modes range (3000‐3700 cm^−1^), employing an optical parametric oscillator/amplifier (OPO/OPA) laser system (LaserVision) pumped by a 10‐Hz Nd:YAG laser which is allowed to interact with the ions trapped in the cell of a Paul ion‐trap (Bruker Esquire 6000) mass spectrometer. The apparatus has been already described in detail.^[56]^ Mass‐selected ions were trapped for 10–40 ms, depending on their abundance, and irradiated for 1 to 3 s on the basis of their fragmentation yield. The OPO/OPA laser system generated an energy of ca. 15 mJ pulse^−1^ with a spectral width of 5 cm^−1^. The IR action spectra are obtained by plotting the photofragmentation yield R=− ln[I_p_/(I_p_ + ΣI_f_)], where I_p_ and ΣI_f_ are the parent and sum of the fragment ion abundancies, respectively, as a function of the radiation wavenumber.^[57]^

### Computational Details

Guess structures of isomers of the protonated Pt‐containing complexes were optimized using the B3LYP functional and the 6–311+G(d,p) basis set for light atoms. For platinum the LanL2TZ basis set and ECP was used. All isomers were reoptimized with the double hybrid B2PLYP functional adding the D3 Grimme dispersion correction method, and the def2TZVP basis set for all atoms.[^58^, ^59^, ^60^, ^61^] Harmonic IR spectra are presented scaled by a factor of 0.955,[^43^, ^45^, ^46^, ^47^, ^61^, ^62^] in agreement with previous works on Pt‐complexes, and convoluted with a Gaussian profile considering a FWHM of 10 cm^−1^. All calculations were performed using Gaussian 16 rev.C01.^[63]^

## Results and Discussion

### Collision‐Induced Dissociation Experiments

**Figure 1:**
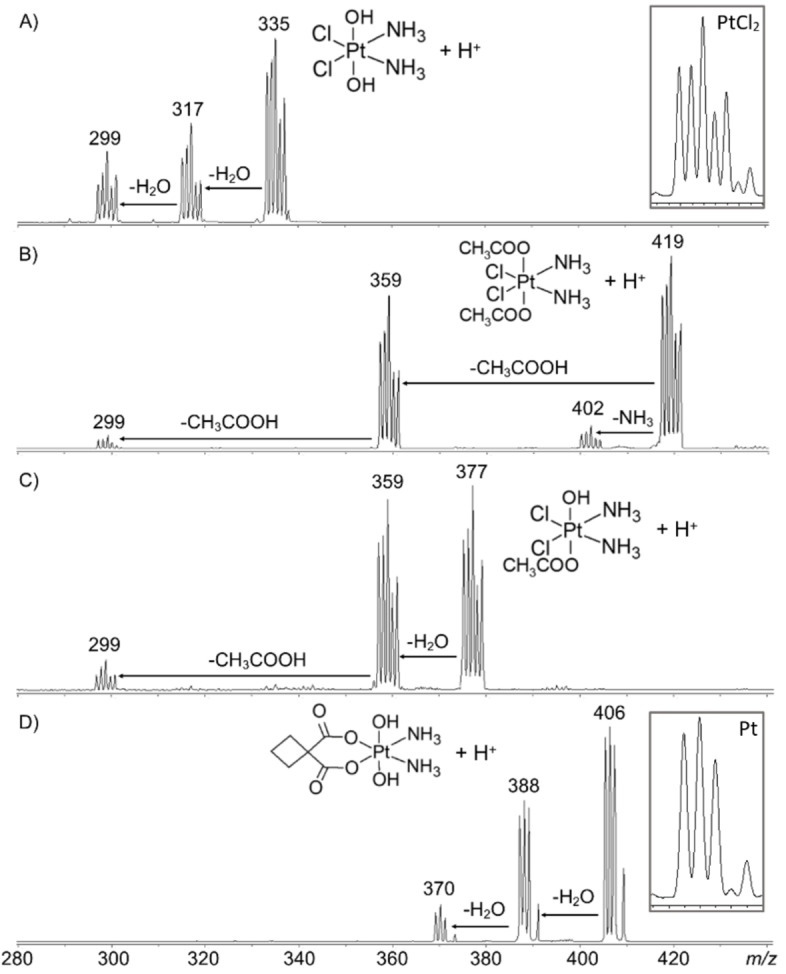
Mass spectra obtained upon mass selection and collisional activation of A) [**1**+H]^+^, B) [**2**+H]^+^, C) [**3**+H]^+^ and D) [**4**+H]^+^. Formal neutral losses are reported. Insets show the calculated isotopic clusters of ions containing either Pt and two Cl atoms or the sole Pt atom. In the case of the fragment of [**1**+H]^+^ centered at *m/z* 299, the isotopic cluster indicates that it retains both chlorine atoms, confirming its attribution to [PtCl_2_N_2_H_5_]^+^. Below *m/z* 280 no fragments were observed or all species.

The protonated complexes [**1**+H]^+^, [**2**+H]^+^, [**3**+H]^+^ and [**4**+H]^+^ were produced by ESI, and their isotopic clusters were mass selected in the cell of a quadrupole ion‐trap (Esquire 6000 plus, Bruker). The five main isotopic peaks were mass‐selected for the species containing Pt and Cl ([**1**+H]^+^ at *m/z* 333–337, [**2**+H]^+^ at *m/z* 417–421 and [**3**+H]^+^ at *m/z* 375–379), while 4 main isotopes are considered in [**4**+H]^+^ (*m/z* 405, 406, 407 and 409) due to the absence of any chlorine atom. When activated by CID, the whole set of protonated compounds primarly dissociates by the loss of a neutral molecule from the axial ligands. The resulting spectra are reported in Figure 1.

As shown in Figure 1, in the cases of the species presenting equal axial ligands, i. e. [**1**+H]^+^, [**2**+H]^+^ and [**4**+H]^+^, the main fragments correspond to the loss of either of these ligands as neutral species; specifically, elimination of H_2_O (product ion at *m/z* 315–319), acetic acid (*m/z* 357–361) and again water (*m/z* 387–391), respectively, followed by the loss of the remaining axial ligands. The consecutive nature of this process is confirmed by CID experiments performed employing a linear ion trap (LTQ‐XL), where consecutive fragmentation events are inhibited. Results are reported in Figure S1. In the specific case of [**2**+H]^+^, the prominent acetic acid dissociation channel is accompanied by ammonia loss (*m/z* 400–404), although showing minor intensity. Therefore, from the CID of the symmetric species, [**1**+H]^+^, [**2**+H]^+^ and [**4**+H]^+^, the dissociation of the axial ligand appears favored. This evidence is in agreement with previous studies on the unimolecular dissociation of satraplatin, *trans,cis,cis*‐diacetatoammine(cyclohexylamine)dichloridoplatinum(IV), the first Pt(IV) compound to have entered phase 3 of clinical trials, which show a predominant dissociation of the axial acetato ligand.^[64]^ Additionally, the case of [**2**+H]^+^, whose fragmentation pattern involves ammonia loss in competition with acetic acid, suggests the acetate group to be more difficult to dissociate in the gas‐phase from platinum as compared to the water molecule. This line of reasoning is confirmed looking at the dissociation of the complex [**3**+H]^+^, which exhibits a mixed situation where the axial positions are occupied by either acetato or hydroxido ligands, as reported in Scheme 1. The CID spectrum of [**3**+H]^+^ (Figure 1 C) shows primary water loss (*m/z* 357–361) eventually followed by the cleavage of acetic acid at *m/z* 297–301. We could not find any evidence of a competing channel involving acetic acid loss directly from the parent ion (Figure S1) confirming the lower dissociation threshold of water compared to acetic acid from Pt(IV). It should be noted that in all the reported fragmentation pathways, the metal atom is not involved in any reduction process. This could be attributed to the ease with which the protonated axial ligands dissociate as neutral species, thereby avoiding redox reactions. In contrast, the same species in their deprotonated form, as well as non‐covalent complexes of Pt(IV) with ascorbate, exhibit a different behavior, generating reduced Pt species by activation in the gas‐phase, as reported in previous studies that explored the gas‐phase reactivity of these complexes.[^25^, ^65^, ^66^]

In the forthcoming section, the vibrational features of mass selected [**1**+H]^+^, [**3**+H]^+^ and [**4**+H]^+^ ions, will be discussed in comparison with theoretically calculated IR spectra in order to unveil their main structural characteristics. Notably, the IRMPD spectrum of [**2**+H]^+^ is not reported due to the absence of any significant fragmentation during the scan of the XH stretching range.

### Vibrational and Structural Features of [1+H]^+^

**Figure 2:**
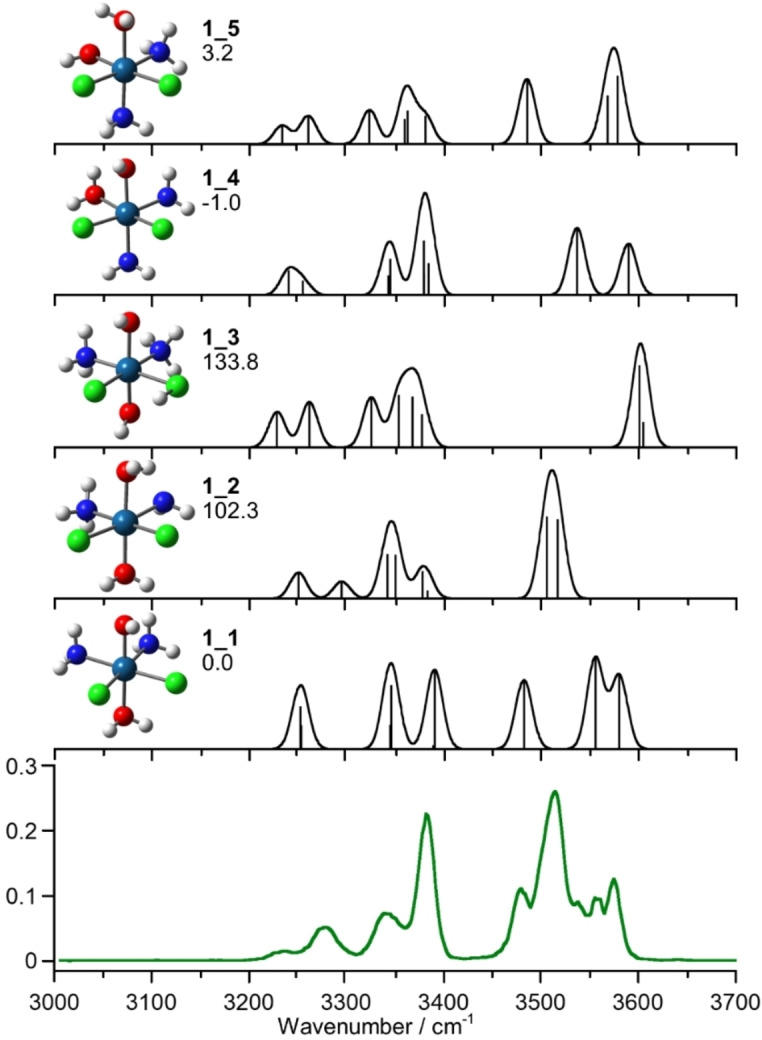
IRMPD spectrum of [**1**+H]^+^ (green profile) compared to the theoretically calculated spectra of the **1**_**1**‐**5** isomers at B2PLYP−D3 level. Geometries are reported on the left side together with Gibbs energies at 298 K in kJ mol^−1^. All calculated spectra are scaled by a factor of 0.955.

In Figure 2, the IRMPD spectrum of [**1**+H]^+^ is reported and compared with the calculated IR spectra of **1** isomers at the B2PLYP level.

The isomers explored as potential structures of [**1**+H]^+^ are reported in Figure 2. They include three structures characterized by protonation on different ligands of the complex (**1**_**1**‐**3**) and two ligand scrambled structures (**1**_**4**,**5**). **1**_**1**, the lowest‐energy protomer of **1**, retains the cisplatin equatorial structure and is protonated on the axial OH group, with the two hydrogens of the so formed water molecule oriented towards the chlorido ligands. **1**_**3**, characterized by protonation on a chlorido ligand, is much higher in relative Gibbs energy, lying at 134 kJ mol^−1^. This energy difference aligns with the higher proton affinity of water compared to HCl (691.0 vs. 556.9 kJ mol^−1^, respectively).^[67]^ Interestingly, an additional prototropic minimum energy structure have been identified, **1**_**2**, featuring protonation on one axial OH ligand and a proton transfer from one ammonia molecule to the remaining OH, thus forming two water molecules bound axially to Pt. This structure is 103 kJ mol^−1^ higher in energy and contains one water molecule oriented toward the chlorido ligands, while the other interacts with the amino group. Structures **1**_**4** and **1**_**5** result from an exchanged position of ammonia and hydroxido ligands. The lowest energy species, **1**_**4**, is protonated on the OH in trans to the chlorido, while **1**_**5** is protonated on the other OH ligand. However, ligand scrambling in octahedral Pt(IV) complexes has not been observed to date,[^27^, ^66^] making such rearrangements unlikely under our experimental conditions.

The experimental vibrational features were compared to the calculated spectra of the reported isomeric structures (Figure 2). The sampled OH stretching range, from 3450 to 3600 cm^−1^, shows a complex cluster of bands indicative of the presence of multiple isomers. Interestingly, the best match is obtained by considering **1**_**1** and **1**_**2**. Accordingly, the experimental IRMPD bands compared with the calculated vibrational modes of the two isomers are reported in Table 1. The convoluted band from 3450 to 3600 cm^−1^ can be assigned in part to the calculated OH stretching mode at 3577 cm^−1^, and to the asymmetric and symmetric stretchings of the water molecule at 3548 and 3478 cm^−1^, respectively, of **1**_**1**. However, the main experimental IRMPD band at 3515 cm^−1^ aligns only with **1**_**2**, where asymmetric stretches of water molecules interacting with the amino group or chlorido ligands occur at 3516 and 3506 cm^−1^, respectively. Four absorptions are recorded at lower wavenumber, namely at 3383, 3340, 3278 and 3240 cm^−1^, that are interpreted by the asymmetric and symmetric stretchings of the ammonia molecules, as reported in Table 1. The presence of **1**_**3** in the sampled ionic population is rather discarded in view of calculated vibrations above 3600 cm^−1^, where no activity is observed. Finally, while **1**_**4** and **1**_**5** could account for some spectral features, the absence of specific experimental bands (e. g., at 3515 cm^−1^) and the unlikelihood of ligand rearrangement rather dismiss their contribution. Moreover ligand scrambling was not observed when the same complex was spectroscopically analyzed as either deprotonated species or non‐covalently bound with ascorbate.[^25^, ^65^, ^66^] In summary, the IRMPD sampling of [**1**+H]^+^ supports the combined presence of **1**_**1** and **1**_**2**. However, the relative contribution of these species cannot be determined because IRMPD signal intensities do not directly correlate with the extinction coefficients of vibrational modes, due to the non‐linear nature of the multiple photon absorption process.[^57^, ^68^, ^69^]

**Table 1:** Experimental IRMPD bands of [**1**+H]^+^ and calculated vibrational modes of **1**_**1** and **1**_**2**.

[1+H]^+^			
Experimental IRMPD^a^	Calculated IR frequencies^a,b^		Vibrational mode assignment
	**1_1**	**1_2**	
3577	3573 (108)		OH stretching
3555	3548 (134)		H_2_O asymm stretching
3515		3516 (173)	H…OH asymm stretching
		3506 (179)	H_2_O asymm stretching
3478	3474 (101)		H_2_O symm stretching
3383	3383 (113)	3378 (58)	NH_3_ asymm stretching
3340	3339 (92)	3350 (95)	NH_3_ asymm stretching
	3338 (34)		NH_3_ asymm stretching
		3342 (96)	H_2_O symm stretching
3278		3295 (38)	NH_2_ symm stretching
3240	3246 (33)	3250 (57)	NH_3_ symm stretching
	3245 (61)		NH_3_ symm stretching
		2807 (172)	H…OH symm stretching

^a^ in cm^−1^. ^b^ Intensities are reported in brackets in km mol^−1^.

The presence of **1**_**2** is particularly intriguing. High‐energy isomers are typically observed in the gas‐phase population of ESI‐generated ions when they are predominantly formed in solution and cannot interconvert in the gas phase due to high transition‐state (TS) energies for conversion.[^70^, ^71^, ^72^] However, the TS located at 100.1 kJ mol^−1^ for the proton transfer from **1**_**2** to **1**_**1**, shows a lower Gibbs energy than **1**_**2**, thus indicating a loose transition state (Figure S2). Contrary to previous assumptions, this evidence suggests that proton transfer should occur readily in the gas‐phase as a barrierless process from **1**_**2** to **1**_**1**. Therefore, the presence of **1**_**2** cannot result from kinetic trapping of this isomer during the ionization process.

Another notable observation concerns the IRMPD mass spectrum in the entire range, which almost exclusively shows a signal corresponding to the simultaneous loss of both water molecules (Figure S3). This outcome contrasts with the CID spectrum, where the first water loss is evident and predominant, accounting for approximately 50 % of the precursor‘s dissociation compared to the loss of the second water molecule (Figure 1). This discrepancy is unexpected, because the IRMPD process generally behaves similarly to CID in terms of ion energy distribution. Here, however, the IRMPD results and the identified spectroscopic features suggest that the presence of bands related to **1**_**2** may be linked to preliminary vibrational activation of **1**_**1** by the initial photons absorbed. This activation could transiently populate the higher‐energy isomer (**1**_**2**), which then readily undergoes dissociation, primarily resulting in the simultaneous loss of both water molecules.

### IRMPD Spectroscopy of [3+H]^+^

**Figure 3:**
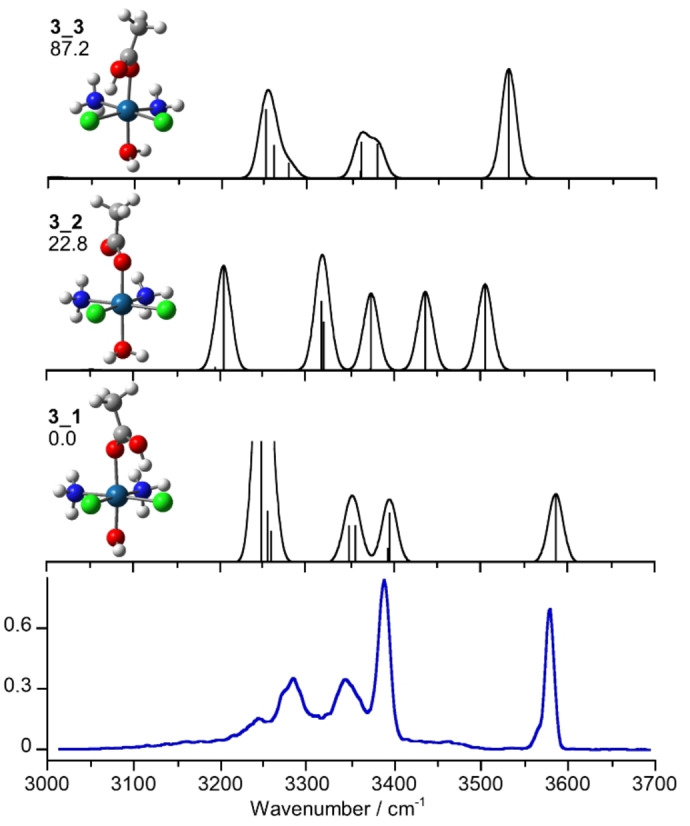
IRMPD spectrum of [**3**+H]^+^ (blue profile) compared to the theoretically calculated spectra of the structures **3**_**1**, **3**_**2** and **3**_**3** at the B2PLYP−D3 level scaled by a factor of 0.955. Geometries are shown on the left together with relative Gibbs energies at 298 K in kJ mol^−1^.

Figure 3 displays the IRMPD spectrum of [**3**+H]^+^ compared to IR spectra of calculated isomers exhibiting protonation on either the acetato (**3**_**1**) or hydroxido (**3**_**2**) ligands. Additionally, a structure with both the acetato and hydroxido ligands protonated was also considered, namely **3**_**3**, as suggested by previous evidence gathered about [**1**+H]^+^. **3**_**1** is the lowest lying isomer and presents a H‐bond involving the OH of the protonated acetato ligand and a chlorine atom. **3**_**2** represents instead the isomer characterized by protonation on the hydroxido ligand. This isomer shows the CO group of the acetate group directed towards the ammonia ligands and is placed at a relative Gibbs energy of 22.8 kJ mol^−1^. Higher‐energy conformers with protonation on the acetato ligand (**3**_**4** and **3**_**5**) and an isomer with the chlorine atom protonated (**3**_**6**) have also been considered and are reported in Figure S4. In particular, the structure **3**_**4**, lying at 19.8 kJ mol^−1^, shows a non‐covalent interaction of a methyl hydrogen with a chlorido ligand, and the carboxylic acid moiety in *cis* conformation, while at 35.9 kJ mol^−1^ the **3**_**5** conformer has the carboxylic function in trans conformation with the oxygen oriented toward one of the hydrogens of the ammonia molecule. Isomer **3**_**6** is the higher in relative energy (118.9 kJ mol^−1^) and show the added proton oriented toward the oxigen of the acetato bound to Pt.

The IR spectrum of **3**_**1** in Figure 3 accounts for all major absorptions. Notably, it shows a single vibrational mode in the high‐frequency region of the spectrum corresponding to the OH stretching calculated at 3578 cm^−1^. This vibration matches well with the experimental band at 3579 cm^−1^. In contrast, **3**_**3** features only one vibrational mode in the high‐frequency region, namely the asymmetric H₂O stretching calculated at 3531 cm^−1^, which does not align with the experimental feature. In the range below 3450 cm^−1^, which is characterized by NH and CH stretching modes, three major bands can be highlighted at 3387, 3346 and 3286 cm^−1^, which are assigned to the ammonia asymmetric and symmetric stretchings calculated for **3**_**1** at 3388, 3348, 3341 and 3251, 3248 cm^−1^, respectively. Additionally, the broad absorption with maximum at 3239 cm^−1^ shows consistency with the OH stretching mode of the carboxilic acid calculated at 3240 cm^−1^. This assignment is consistent with the fact that the H atom is engaged in a H‐bond with the chlorido ligand. In fact, such vibrational motifs, particularly stretching motions involving hydrogen‐bonded atoms, tend to produce broadened IRMPD bands. This broadening arises from the asymmetric nature of the vibration, which disrupts the multiple‐photon absorption process.[^68^, ^69^, ^73^, ^74^] The experimental bands and calculated vibrational modes for the two selected isomers **3**_**1** and **3**_**2** are reported in Table 2. It is evident that **3**_**2** does not significantly contribute to the sampled gas‐phase population. This conclusion is supported by the calculated wavenumbers of its characteristic asymmetric and symmetric H₂O stretching modes at 3505 and 3437 cm^−1^, which fall in a region of the spectrum devoid of significant experimental absorptions.

**Table 2:** Experimental IRMPD bands of [**3**+H]^+^ and calculated vibrational modes of **3**_**1** and **3**_**2**.

[3+H]^+^			
Experimental IRMPD^a^	Calculated IR frequencies^a,b^		Vibrational mode assignment
	**3_1**	**3_2**	
3579	3578 (114)		OH stretching
‐		3505 (142)	H_2_O asymm stretching
‐		3437 (130)	H_2_O symm stretching
3387	3388 (82)	3374 (126)	NH_3_ asymm stretching
3346	3348 (61)	3319 (79)	NH_3_ asymm stretching
	3341 (60)	3317 (114)	NH_3_ asymm stretching
3286	3251 (51)		NH_3_ symm stretching
	3248 (85)		NH_3_ symm stretching
3239	3240 (429)	3204 (171)	CO−H stretching

^a^ in cm^−1^. ^b^ Intensities are reported in brackets in km mol^−1^.

### IRMPD Spectroscopy of [4+H]^+^

**Figure 4:**
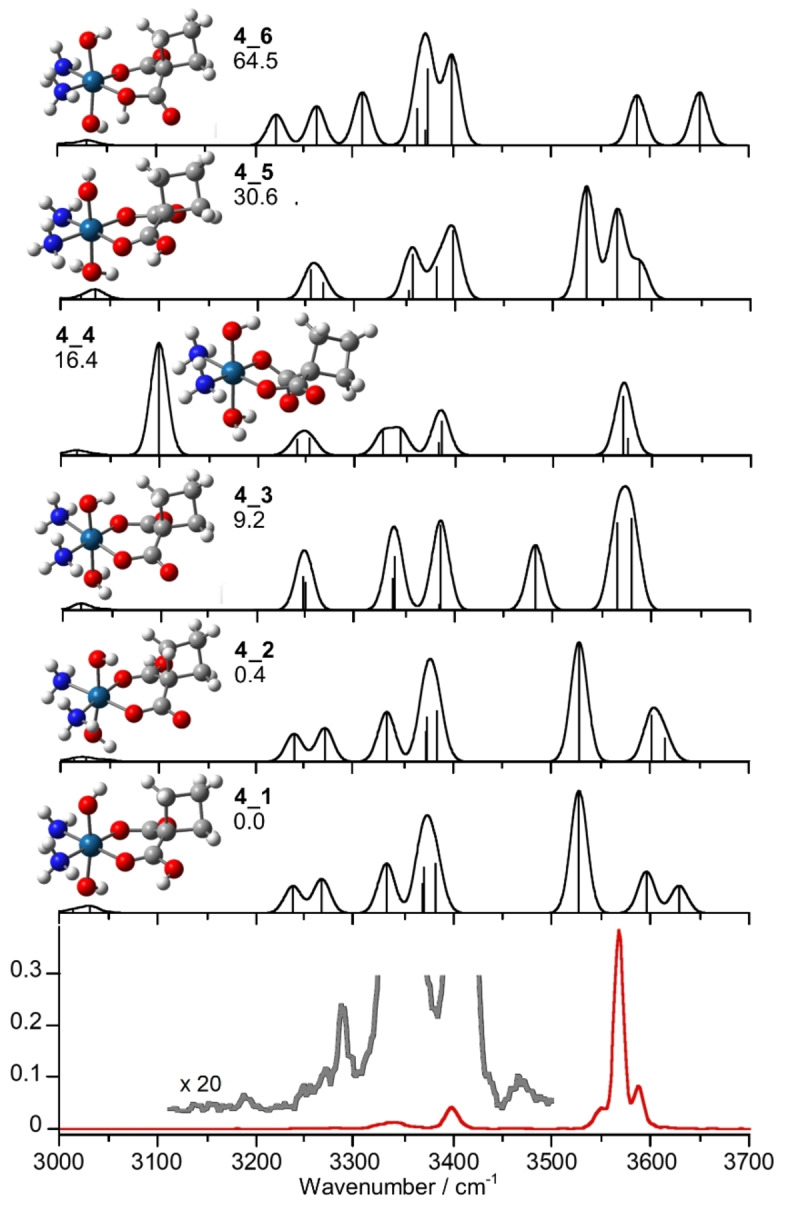
IRMPD spectrum of [**4**+H]^+^ (red and grey profiles) compared to the theoretically calculated spectra of the structures **4**_**1**‐**6** at the B2PLYP−D3 level scaled by a factor of 0.955. Geometries are shown on the left together with relative Gibbs energies at 298 K in kJ mol^−1^.

Complex **4** contains two axial hydroxido ligands and a cyclobutane‐1,1‐dicarboxylate chelating ligand in equatorial position, together with two ammonia molecules. Two conceivable protonation sites can involve the axial hydroxido ligand or the carboxylate moiety of the chelating ligand. Figure 4 illustrates the optimized geometries of selected calculated isomers along with their theoretical IR spectra, together with the experimental IRMPD spectrum. The most stable structures, **4**_**1** and **4**_**2**, are both protonated on one or the other carboxylate oxygen atoms not bound to Pt, in agreement with the ascribed structure of protonated carboplatin.^[43]^ In both cases, the proton is oriented toward the other oxygen of the same carboxylate group in a cis‐like configuration. Protonation on the latter oxygen results in an isomer, **4**_**6**, which lies 64.5 kJ mol^−1^ higher in energy relative to **4**_**1**. This energy gap is consistent with the increased acidity of the COH group due to the umpolung effect induced by its binding to platinum. The structures of **4**_**3** and **4**_**4** are both protonated on the axial OH, but differ in the positioning of the carboxylate groups of the cyclobutane‐1,1‐dicarboxylate ligand relative to the protonation site. Notably, **4**_**3** presents a lower energy than **4**_**4**, lying at 9.2 kJ mol^−1^, and exhibits a quasi‐planar geometry for the five‐membered ring formed by the carboxylato groups and the platinum atom. In this structure, the protonated hydroxo ligand and the cyclobutyl group occupy opposite sides relative to the plane defined by the five‐membered ring. Conversely, **4**_**4** shows a H‐bond interaction involving the H‐atom of the aqua ligand and the carboxylate oxygen (Figure 4). Despite this additional intramolecular interaction, the structure is higher in energy, lying at 16.4 kJ mol^−1^. The reduced stability likely arises from the steric strain imposed on the cyclobutane‐1,1‐dicarboxylate ligand by the H‐bond interaction. Finally, **4**_**5** (30.6 kJ mol^−1^) explores the possibility of a proton transfer from the ammonia molecule to water starting from the global minimum **4**_**1**.

The spectroscopic evidence aligns with the computed relative energies, showing a fair agreement between the IRMPD spectrum and the calculated spectra of the lowest energy isomers protonated on either the carboxylato ligands (**4**_**1** and **4**_**2**) or the hydroxido ligand (**4**_**3**). The experimental features together with the calculated ones for the selected structures are reported in Table 3. Of particular interest is the range of the OH stretching modes at 3500–3650 cm^−1^, which shows three experimental bands, suggesting the presence of a mixed population. The intense IRMPD band at 3569 cm^−1^ is well simulated by OH stretching modes of the hydroxido and aqua ligands, calculated for **4**_**3** at 3580 and 3567 cm^−1^, respectively. The absorptions corresponding to the axial OH stretching modes of **4**_**1** and **4**_**2** predicted at 3630 and 3613, and at 3596 and 3600 cm^−1^, respectively, could account for the experimental band at 3586 cm^−1^. Additionally, the COH stretching mode of **4**_**1** and **4**_**2**, calculated at ca. 3527 cm^−1^, accounts for the experimental band observed at 3545 cm^−1^. **4**_**3** also exhibits a vibration at 3483 cm^−1^ that could explain the 3463 cm^−1^ experimental band, although this mode shows limited IRMPD activity.[^75^, ^76^] Absorptions in the spectral range below 3400 cm^−1^ can be attributed to a combination of the NH stretchings of the ammonia molecules, calculated in this range for the representative isomers **4**_**1**, **4**_**2** and **4**_**3**.

**Table 3:** Experimental IRMPD bands of [**4**+H]^+^ and calculated vibrational modes of **4**_**1**, **4**_**2** and **4**_**3**.

[4+H]^+^				
Experimental IRMPD	Calculated IR frequencies			Vibrational mode assignement
	**4_1**	**4_2**	**4_3**	
3586	3630 (40)	3613 (33)		OH stretching
	3596 (60)	3600 (67)	3580 (133)	OH stretching
3569			3567 (127)	H_2_O asymm stretching
3545 (shoulder)	3528 (178)	3526 (173)		CO−H stretching
3463			3483 (94)	H_2_O symm stretching
3397	3382 (72)	3381 (73)	3387 (122)	NH_3_ asymm stretching
	3370 (66)	3371 (64)		NH_3_ asymm stretching
	3369 (43)	3370 (43)		NH_3_ asymm stretching
3339	3332 (72)	3345 (74)	3340 (77)	NH_3_ asymm stretching
			3338 (45)	NH_3_ asymm stretching
3281	3267 (49)	3268 (49)	3250 (40)	NH_3_ symm stretching
	3237 (39)	3237 (40)	3247 (48)	NH_3_ symm stretching

^a^ in cm^−1^. ^b^ Intensities are reported in brackets in km mol^−1^.

Spectroscopically, **4**_**4** and **4**_**5** could participate to the gas‐phase population, albeit as minor components. It is worth noting that the OH stretching mode calculated for **4**_**4** at ~3100 cm^−1^, associated with the H‐bonded hydroxido group, could be IRMPD transparent possibly due to the disruption of the H‐bond network produced by the first photon absorption.[^69^, ^75^, ^76^] Finally, the spectrum of **4**_**6** does not match the experiment, and any significant presence of this isomer can be excluded.

To summarize, the assayed gas‐phase population mainly consists of **4**_**3, 4**_**1** and **4**_**2**, with a slight predominance of the isomer protonated on the hydroxido ligand (**4**_**3**). The reported attributions are also confirmed by comparison of the calculated and experimental IR spectra in the fingerprint region, recorded for [**4**+H]^+^ to aid in solving the complexity of its ionic population and reported in Figure S5.

### Discussion

**Figure 5:**
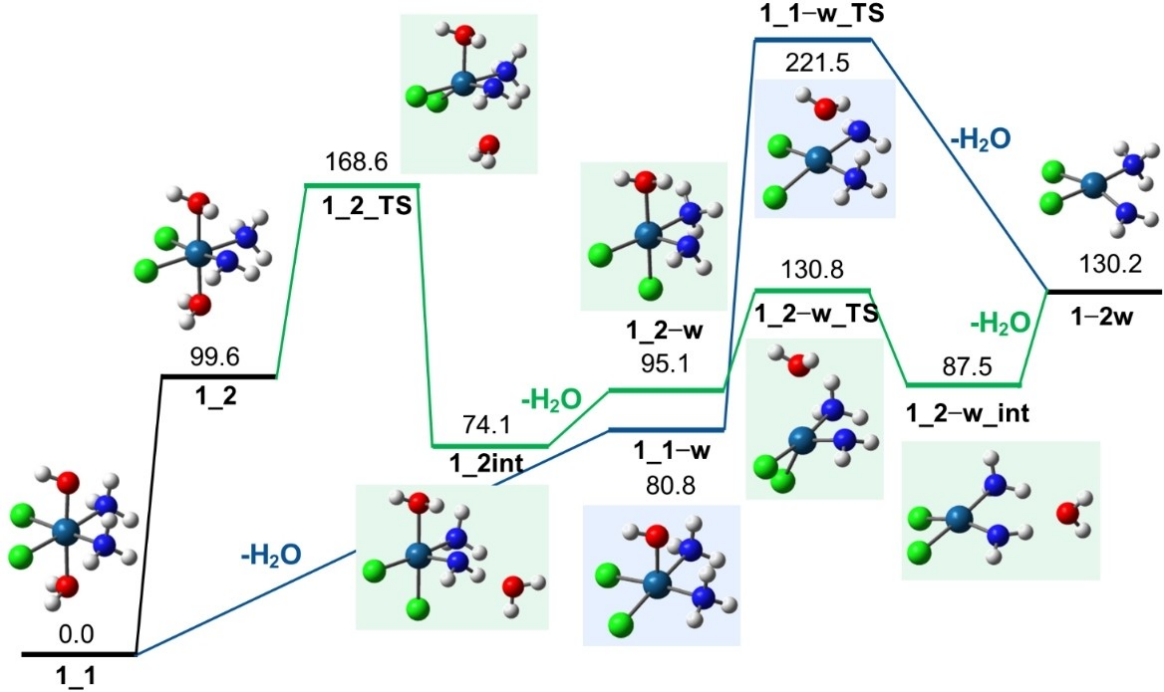
PESs for the water dissociation reaction from **1**_**1** (blue profile) and **1**_**2** (green profile). Optimized structures at the B3LYP level are shown together with relative Gibbs energies at 298 K in kJ mol^−1^.

IRMPD spectroscopy has allowed to characterize the protonated complexes of **1**, **3** and **4**, ionic species formed in solution and brought to the gas‐phase using an ESI source. [**1**+H]^+^ displays protonation on the hydroxido axial ligand, in agreement with its fragmentation behavior which involves a facile cleavage of water. Additionally, its IRMPD spectrum reveals the presence of a high energy species with two aqua ligand in the axial positions and one of the ammonia molecules turned into an amido ligand, NH_2_. Interestingly, the presence of this species appears to be enhanced during the IRMPD process considering the different fragmentation pattern of CID compared to IRMPD. In fact, a consecutive loss of two water molecules is observed under CID while the concurrent departure of both water molecules occurs in the process of IRMPD. The energetic of the process has been investigated by calculations at the B3LYP/6‐311+G(d,p) (Pt=LanL2TZ) level. Two possible scenarios were considered: 1) direct cleavage of water from **1**_**1** followed by proton transfer from ammonia to the remaining hydroxido ligand and finally dissociation of the second water molecule; 2) sequential cleavage of both water molecules from **1**_**2**. Figure 5 reports the potential energy surfaces (PESs) for the two processes.

The direct cleavage of water from **1**_**1** (blue profile in Figure 5) is calculated to require 80.8 kJ mol^−1^. This process is thermodynamically favored compared to the dissociation of ammonia, which requires 143.3 kJ mol^−1^ at the same level of theory (Figure S6). The product of the water loss (**1**_**1‐w**) must overcome an energetically demanding proton transfer from one of the ammonia molecules to the hydroxido ligand (**1**_**1−w**_**TS**, lying at 221.5 kJ mol^−1^) to enable the dissociation of the second water molecule  (**1−2 w**). The calculated energetics for this fragmentation pathway are in line with the CID results (Figure 1 and Figure S1), where only one water molecule is preferentially dissociated. On the other hand, when submitted to IR photoactivation, the gas‐phase population was found to consist of both **1**_**1** and **1**_**2**, a higher‐energy isomer with a Gibbs energy of 99.6 kJ mol^−1^ at the B3LYP level of theory. This species contains two water molecules, with one of the ammonia molecules already deprotonated  (Figure 2). The green profile in Figure 5 shows that the dissociation of the first neutral water is more energy‐demanding than that from **1**_**1**, due to the presence of a transition state lying at 168.6 kJ mol^−1^ (**1**_**2**_**TS**), where H_2_O begins to depart from the complex while a chlorido ligand takes its place. The following dissociation of the second molecule of water requires only 130.8 kJ mol^−1^, thus making this dissociation pathways to reach dissociation of both water molecules collectively less energetically demanding than the one starting from **1**_**1**. This simulated unimolecular reactivity agrees with the experimental observations, eventually explaining why the IRMPD process, in which the population of **1**_**2** is significant, produces a much lower yield of single water loss compared to the loss of both water molecules (Figure S3).

Calculations at the B3LYP level were employed to characterize also the unimolecular reactivity of [**2**+H]^+^. In particular, the goal was to evaluate why the dissociation of the ammonia molecule was observed exclusively for this complex. As shown in Figure S7, the energy required for the dissociation of acetic acid from the protonated complex **2**_**1** (119.1 kJ mol^−1^) is comparable to the lowest energy needed for the loss of ammonia, corresponding to a transition state located at 121.9 kJ mol^−1^. Direct dissociation of ammonia, leading to a pentacoordinated Pt(IV) complex, is energetically more demanding (156.5 kJ mol^−1^). However, the acetato ligand is able to chelate platinum, forming a more stable structure (**2**_**1‐NH_3_2**) at 79.3 kJ mol^−1^ after overcoming the aforementioned TS. These calculations clearly explain why the dissociation of the ammonia molecule is observed specifically for the protonated complex **2**.

**Scheme 2:**
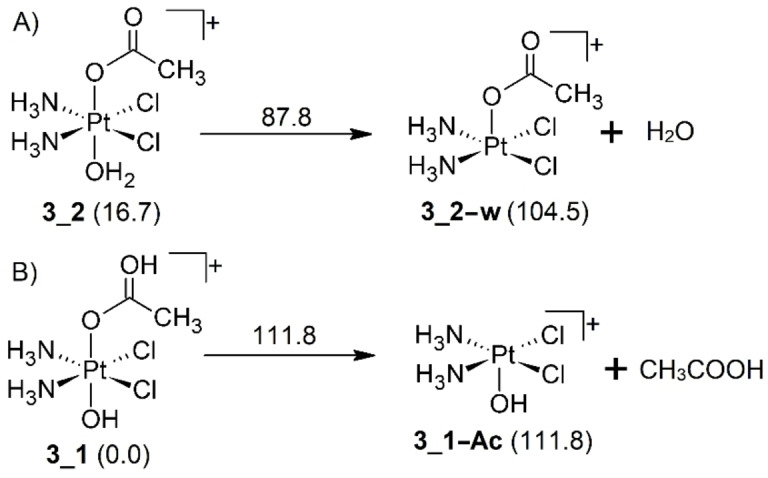
Schematic representation of the direct cleavage of A) water and B) acetic acid from isomers of [**3**+H]^+^ from either the protonated hydroxido ligand (**3**_**2**) or the protonated acetato ligand (**3**_**1**), path A and B, respectively. Relative Gibbs energies of the isomers are reported in parentheses, while the dissociation energy for each process is reported on top of the arrows. All Gibbs energies at 298 K are in kJ mol^−1^.

Regarding protonated complex **3**, its spectroscopic characterization appears to be more straightforward, unveiling a gas‐phase population mostly composed of the lowest lying calculated structure (**3**_**1**), which exhibits protonation on the acetate moiety. Interestingly, the IRMPD mass spectra in the whole IR range explored show a dissociation pattern involving only water loss, as also evidenced by CID experiments (Figure 1). This result suggests that either an isomerization reaction from **3**_**1** to **3**_**2** or a proton transfer from the ammonia to the hydroxido ligand should occur. Energetically, we can expect either one of the two processes to be favored with respect to the direct cleavage of acetic acid, which should be associated to a higher energy threshold. Calculations at the B3LYP/6‐311+G(d,p) (Pt=LanL2TZ) level were employed to evaluate the thermodynamic features of the dissociation process. Scheme 2 reports the calculated Gibbs energies at 298 K for the direct cleavage of either water (A) or acetic acid (B) starting from the isomers of the [**3**+H]^+^ complex that are protonated on either of the axial ligands involved in the neutral loss upon CID, i. e. **3**_**2** and **3**_**1**, respectively.

The dissociation of water from **3**_**2** is calculated to be less endoergonic than the cleavage of acetic acid from **3**_**1**, in agreement with the fact that acetic acid fragmentation is not observed in the first dissociation event (see Figure 1 and Figure S1). At the same time, **3**_**2** is higher in energy than **3**_**1** and IR ion spectroscopy has determined its absence from the ionic population, therefore it is necessary to evaluate the energy required for isomer interconversion. Alternatively, one can hypotesize a proton transfer from ammonia to the axial hydroxido ligand as possible mechanism for water loss from **3**_**1**.

**Figure 6:**
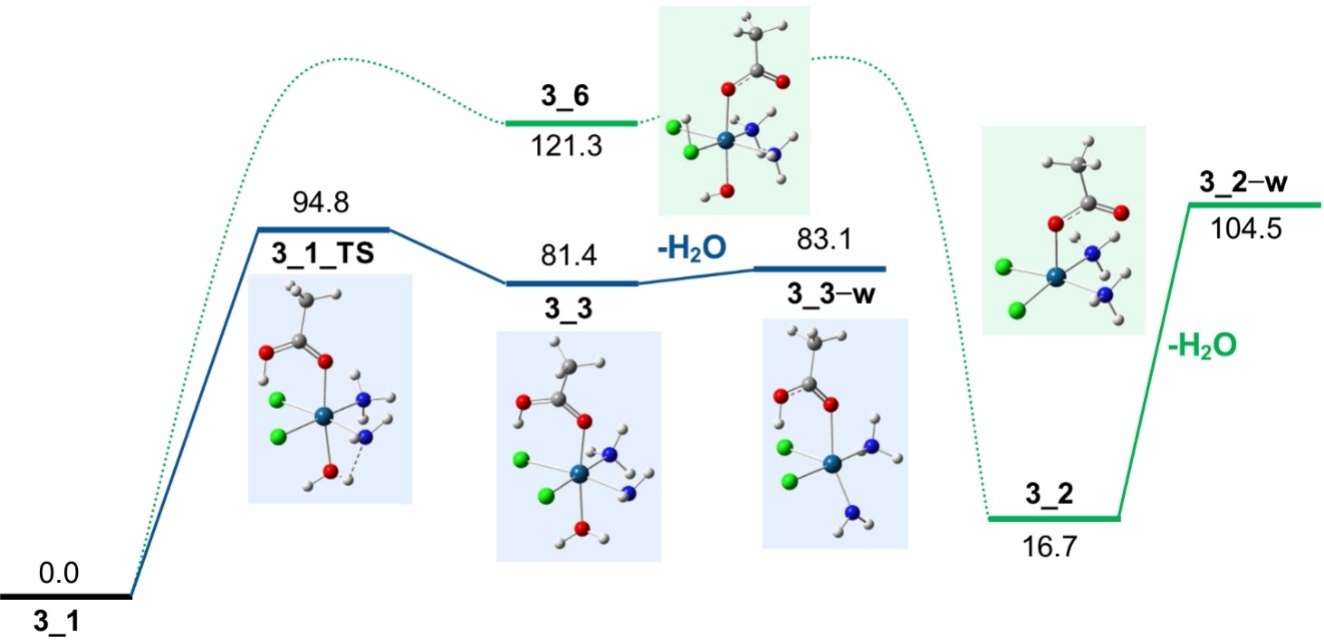
PESs for the water dissociation reaction from **3**_**1**. The blue profile represents the reaction involving proton transfer from the ammonia ligand, while the green profile the isomerization reaction in which the proton is transferred from the carboxilato to the hydroxido ligand through the chlorido. Optimized structures at the B3LYP level are shown together with relative Gibbs energies at 298 K in kJ mol^−1^.

Employing DFT at the B3LYP level, calculations of the energy profile involved in the two mechanisms has been evaluated. The blue profile in Figure 5 shows the PES for the dissociation reaction of water from **3**_**1** that involves a preliminary proton transfer from the ammonia ligand. The activation threshold is given by the energy of the transition state involved in the transfer of the proton from ammonia (**3**_**1**_**TS**) lying at 94.8 kJ mol^−1^ with respect to **3**_**1**. The reaction proceeds via the intermediate characterized by protonation on the hydroxo group (**3**_**3**, 81.4 kJ mol^−1^) followed by a direct cleavage of water from this species accompanied by a change in the overall geometry of the remaining complex **3**_**3−w** as shown in Figure 6.

For the isomerization reaction (green profile, Figure 6), we were unable to locate the transition states corresponding to the proton transfer from the acetate group to the chloride and from the chloride to the hydroxido group. Nevertheless, the first step ultimately results in the intermediate structure **3**_**6**, as shown in Figure 6, which lies at 121.3 kJ mol^−1^. This energy is significantly higher than the activation energy calculated for the proton transfer reaction involving the ammonia ligand (94.8 kJ mol^−1^), indicating that this isomerization mechanism likely plays a negligible role in the water dissociation reaction.

Interestingly, the product resulting from the direct cleavage of water from **3**_**2**, denoted as **3**_**2−w** in Figure 6 and exhibiting a square‐based pyramidal geometry, is higher in energy than its protomer **3**_**3−w**, in which the NH₂ group is displaced from the equatorial plane.

The activation energy for the proton transfer leading to water dissociation (94.8 kJ mol^−1^) was compared to that for the direct cleavage of acetic acid from **3**_**1** (111.8 kJ mol^−1^). The absence of CH₃COOH neutral loss in the fragmentation pattern of [**3**+H]^+^ under either CID or IRMPD conditions is well supported by these calculations, which show that water loss is energetically favored.

A similar analysis applies to [**4**+H]^+^, where two protomers, **4**_**1** (protonated on the free carboxylate oxygen of the cyclobutane‐1,1‐dicarboxylate) and **4**_**3** (protonated on the axial hydroxido ligand), may contribute to the gas‐phase population. Notably, IRMPD bands characteristic of either isomer show the same dissociation path, leading to loss of water. Also in this case we can assume that dissociation occurs after a proton transfer from the ammonia molecule, generating the intermediate isomer **4**_**4**, endowed with an even lower relative Gibbs energy when compared to **3**_**3**, i. e. 30.6 and 80.2 kJ mol^−1^ at the B2PLYP level, respectively.

## Conclusions

Collision‐induced dissociation (CID) experiments on various protonated Pt(IV) complexes with antineoplastic activity have demonstrated that when at least one axial hydroxido ligand is present, water dissociation becomes the primary fragmentation pathway. However, structural characterization of the complexes [**3**+H]^+^ and [**4**+H]^+^ has revealed a significant portion of the gas‐phase population corresponds to isomers protonated on the carboxylato moieties located in either axial or equatorial positions, respectively.

Calculations indicate that water dissociation occurs following a preliminary proton transfer from the ammonia ligand. This mechanism is further supported by the analysis of [**1**+H]^+^, a symmetric complex featuring two hydroxido ligands. In [**1**+H]^+^, the proton transfer activates IR bands associated with a high‐energy isomer possessing two aqua ligands in the axial positions.

Given the critical role of prototropic equilibria in influencing the activity of platinum‐containing drugs, it could be relevant to determine how charges are distributed among the axial and equatorial ligands. Notably, Pt(IV) complexes are being investigated as potential orally available alternatives to cisplatin and related compounds. Upon oral administration, these complexes would encounter the acidic environment of the stomach, where hydrolysis of both axial and equatorial ligands could be accelerated, potentially interfering with the intended activation mechanism which involves reduction within cancer cells.[^31^, ^32^, ^33^, ^35^, ^36^]

These results contribute to the understanding of protonation equilibria and ligand‐specific reactivity of Pt(IV) complexes. We hope these insights may aid in the design of platinum complexes with antineoplastic activity by clarifying their likely forms in acidic environments.

## Conflict of Interests

The authors declare no conflict of interest.

## Supporting information

As a service to our authors and readers, this journal provides supporting information supplied by the authors. Such materials are peer reviewed and may be re‐organized for online delivery, but are not copy‐edited or typeset. Technical support issues arising from supporting information (other than missing files) should be addressed to the authors.

Supporting Information

## Data Availability

The data that support the findings of this study are available from the corresponding author upon reasonable request.
