# Supplementary material for: Protonation of Pt(IV) Anticancer Complexes Assayed by Vibrational Ion Spectroscopy
Source: Chempluschem. 2025 Apr 3;90(6):e202400754. doi: 10.1002/cplu.202400754 (PMC12143457; doi:10.1002/cplu.202400754)
Supplement: Supplementary file 1 — Supporting Information [file CPLU-90-e202400754-s001.pdf]

ChemPlusChem

Supporting Information

Protonation of Pt(IV) Anticancer Complexes Assayed by Vibrational Ion Spectroscopy

Davide Corinti,* Elisabetta Gabano, Barbara Chiavarino, Maria Elisa Crestoni, Domenico Osella, and Simonetta Fornarini

Table of contents

Figure S1. Mass spectra obtained upon mass selection and collisional activation of A) [**1**+H]$^+$, B) [**2**+H]$^+$, C) [**3**+H]$^+$ and D) [**4**+H]$^+$ in a linear ion trap (LTQ-XL) coupled with an ESI source.

Figure S2. Structure and relative free energy of the TS located between **1_1** and **1_2**.

Figure S3. Photofragmentation spectrum at 3380 cm^{-1} of [**1**+H]$^+$.

Figure S4. IRMDP spectrum of [**3**+H]$^+$ (blue profile) compared to the theoretically calculated spectra of the structures **3_4**, **3_5** and **3_6** at the B2PLYP-D3 level.

Figure S5. IRMDP spectrum of [**4**+H]$^+$ in the fingerprint range (red profile) compared to the theoretically calculated spectra of the structures **4_1-_6** at the B2PLYP-D3 level.

Figure S6. Optimized structure at the B3LYP level of the complex produced by dissociation of ammonia from structure **1_1**.

Figure S7. Schematic representation of the direct cleavage of ammonia and acetic acid from **2_1**. Structures optimized at the B3LYP level.

Cartesian coordinates of optimized structures

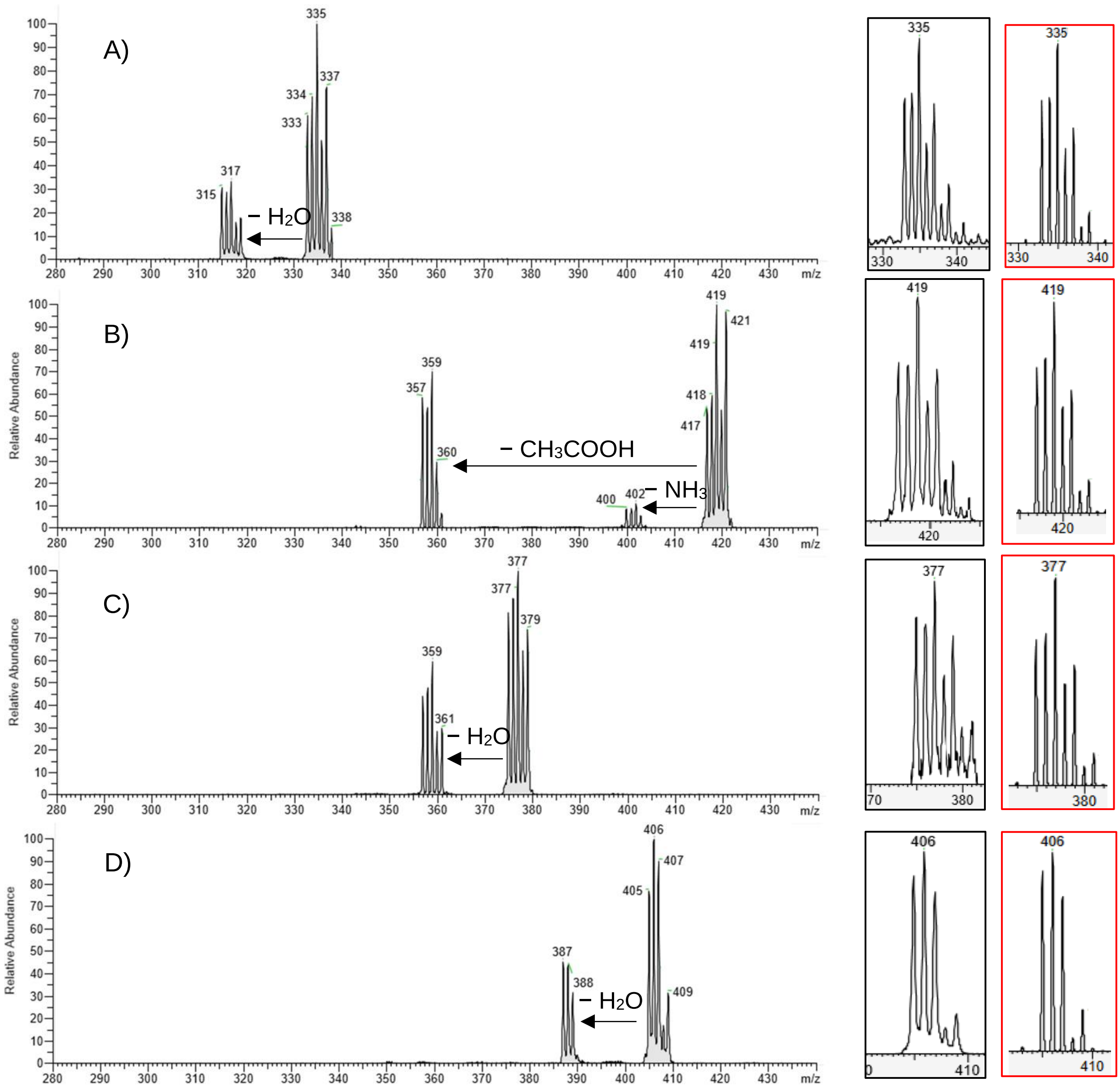

Figure S1. Mass spectra obtained upon mass selection and collisional activation of A) [**1**+H]$^+$, B) [**2**+H]$^+$, C) [**3**+H]$^+$ and D) [**4**+H]$^+$ in a linear ion trap (LTQ-XL by Thermo Fisher Scientific) coupled with an ESI source at a collision eneergy value of 2 V. Formal neutral losses are reported. Deviations in the isotopic cluster from the expected profile are attributed to the selective activation of the first ions in the cluster. On the right, the isotopic cluster recorded without activation energy (to preserve the isotopic profile, shown in black) is compared with the calculated isotopic profile for the corresponding molecular formula (red inset). Below *m/z* 280 no fragments were observed or all species.

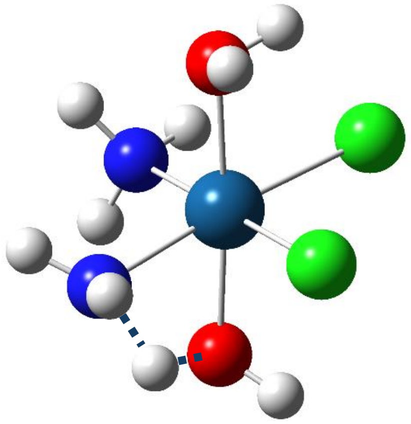

1_TS Rel. G (298K) = 100.1 kJ mol^{-1}

Figure S2. Structure and relative free energy of the TS located between **1_1** and **1_2**

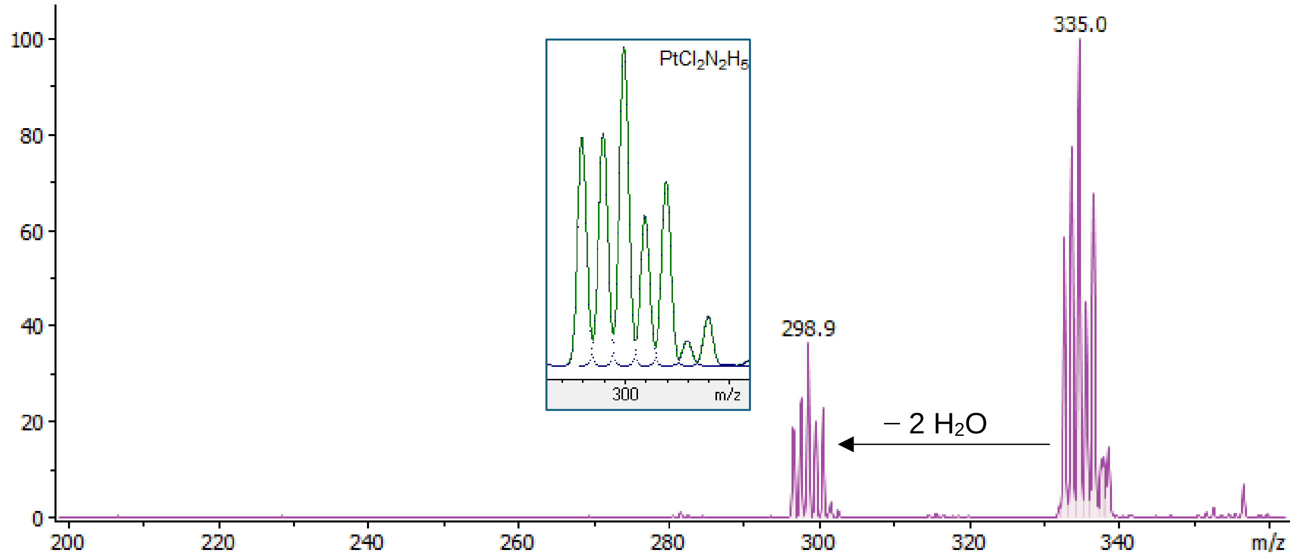

Figure S3. Photofragmentation spectrum at 3380 cm^{-1} of [**1**+H]$^+$. The contemporary dissociation of two molecules of water is confirmed by the isotopic distribution of the fragment which agrees with the theoretical isotopic pattern of an ion presenting two chlorine atoms (green profile in the inset).

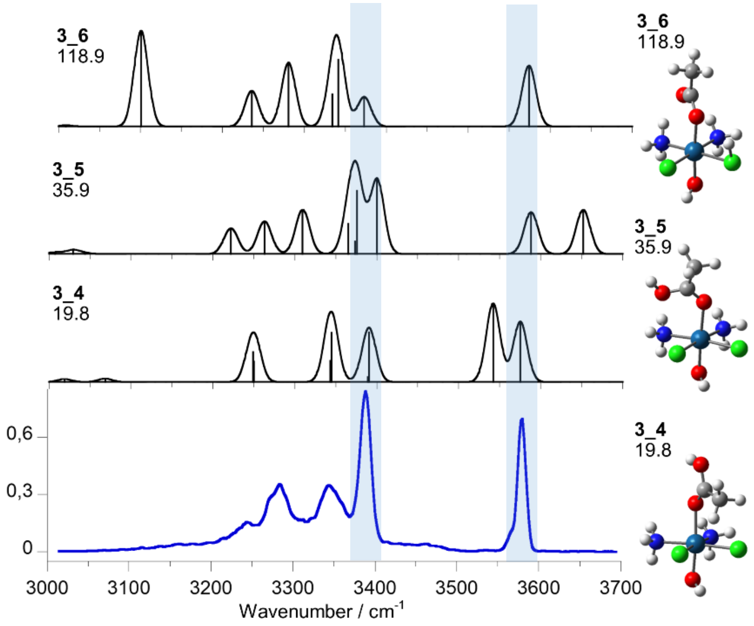

Figure S4. IRMPD spectrum of [**3**+H]$^+$ (blue profile) compared to the theoretically calculated spectra of the structures **3_4**, **3_5** and **3_6** at the B2PLYP-D3 level. Geometries are shown on the right together with relative Gibbs energies at 298K in kJ mol^{-1}.

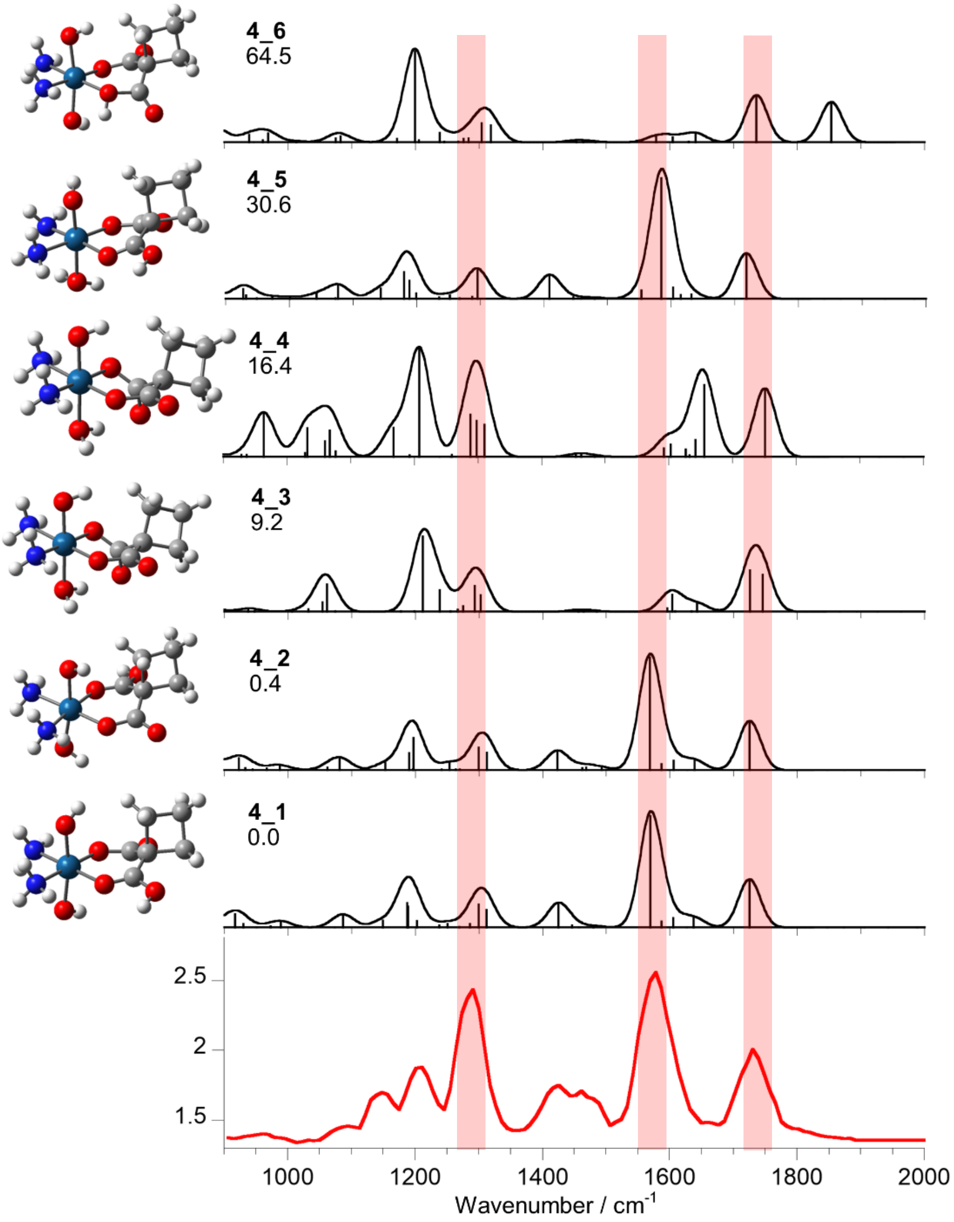

Figure S5. IRMPD spectrum of [**4**+H]$^+$ in the fingerprint range (red profile) compared to the theoretically calculated spectra of the structures **4_1**-**_6** at the B2PLYP-D3 level. Geometries are shown on the right together with relative Gibbs energies at 298K in kJ mol^{-1}. The IRMPD spectroscum in the fingerprint region of the IR spectrum (900-2000 cm^{-1}) was recorded using the free-electron laser (FEL) of the Centre Laser Infrarouge d'Orsay (CLIO). The FEL beamline (operated at 44 MeV for the present experiments) was coupled with a hybrid Fourier transform-ion cyclotron resonance (FT-ICR) tandem mass spectrometer (APEX-Qe Bruker Daltonics), equipped with a 7.0 T actively shielded magnet and a quadrupole-hexapole interface allowing to mass select and accumulate ions prior to irradiation. Calculated spectra are scaled by a factor of 0.974.

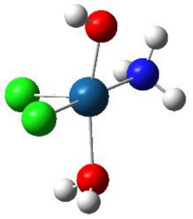

1_1–NH_3 + NH_3

143.3 kJ mol^{-1}

Figure S6. Optimized structure at the B3LYP level of the complex produced by dissociation of ammonia from structure **1_1**. Relative Gibbs energies at 298 K are reported.

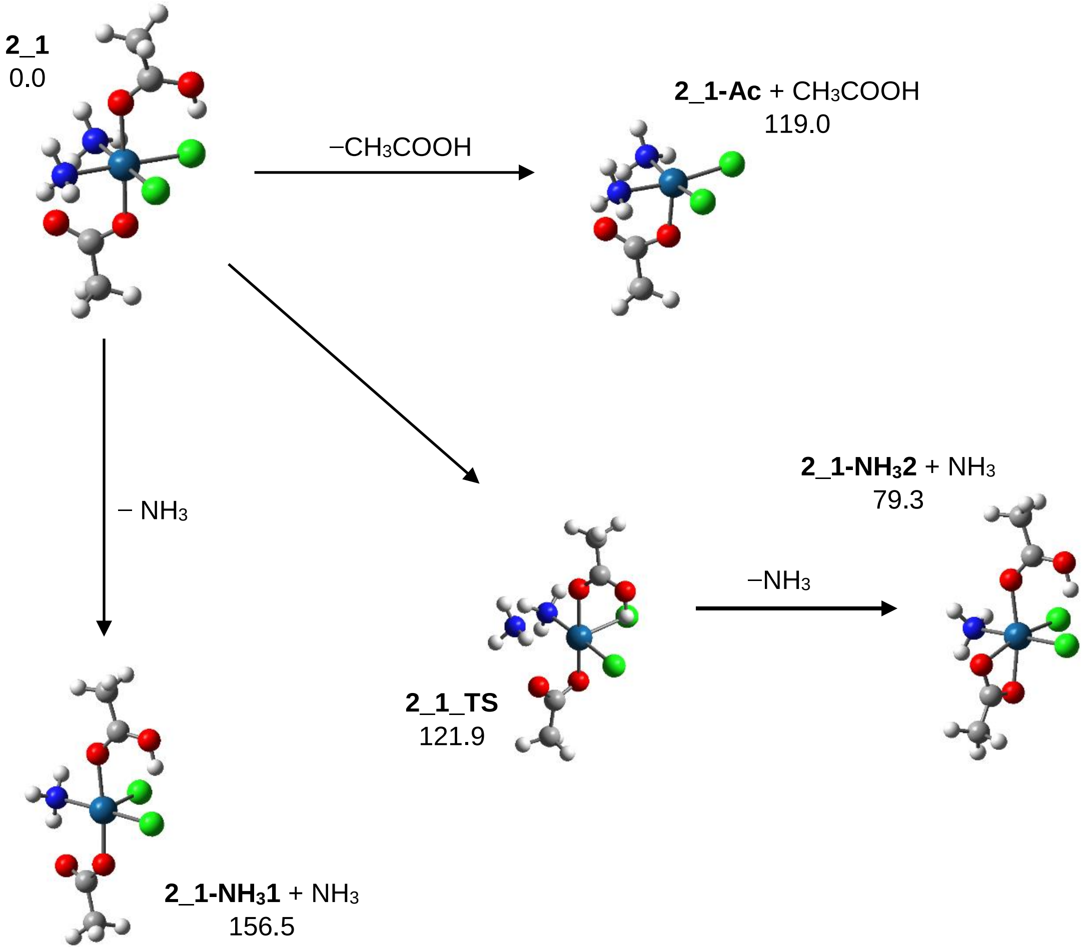

Figure S7. Schematic representation of the direct cleavage of ammonia and acetic acid from **2_1**. Structures optimized at the B3LYP level. All Gibbs energies at 298K are in kJ mol^{-1}.

Cartesian coordinates of Optimized Geometries

Level of theory: B2PLYP-D3/def2-TZVP. Electronic energies (in Hartree/particle) are reported in parentheses. None of the calculated structures exhibit imaginary frequencies.

1_1
(-1303.900268)

Pt	-0.00861700	0.16777100	0.00000000
N	-0.05354500	1.58983200	1.55457400
H	0.15255900	1.10915800	2.43092700
H	-1.02710400	1.89690400	1.59944100
H	0.55705800	2.39989400	1.47781000
N	-0.05354500	1.58983200	-1.55457400
H	-1.02710400	1.89690400	-1.59944100
H	0.15255900	1.10915800	-2.43092700
H	0.55705800	2.39989400	-1.47781000
Cl	-0.05354500	-1.40635900	-1.66379000
Cl	-0.05354500	-1.40635900	1.66379000
O	2.12552700	-0.08363100	0.00000000
H	2.34756800	-0.63351800	-0.77366400
O	-1.93063600	0.49531600	0.00000000
H	-2.37698900	-0.36597200	0.00000000
H	2.34756800	-0.63351800	0.77366400

1_2
(-1303.858764)

Pt	-0.03073700	0.16066400	0.04844700
N	-1.63694000	1.36620800	0.43641500
H	-2.41722900	0.80377500	0.77099400
H	-1.44885800	2.06456300	1.15216300
N	1.30952100	1.78057800	0.21572700
H	1.01060300	2.57840500	-0.34322000
H	2.21033400	1.45869300	-0.14360000
H	1.44421400	2.09646800	1.17462600
Cl	1.84614700	-1.11733100	-0.60048400
Cl	-1.48750400	-1.59135600	-0.14132600
O	0.47375800	-0.51358700	1.91109300
H	1.22676200	-1.12149000	1.72988700
O	-0.58767700	0.90163700	-1.74169400
H	-0.87604300	0.19805100	-2.35240600
H	-0.25115200	-1.06598700	2.26077800
H	-1.39481000	1.37153800	-1.33748700

1_3
(-1303.839220)

Pt	0.16308500	-0.09218500	0.00108000
N	2.11867800	0.34674200	-0.00010800
H	2.20793300	1.36509600	-0.01175600
H	2.55227100	-0.02552400	-0.84653200
H	2.54785200	-0.00569700	0.85707000
N	0.51507300	-2.15961300	0.00654800
H	0.14784000	-2.55324100	-0.86028900

H	0.05700900	-2.57607500	0.81699400
H	1.49936200	-2.41154300	0.06137900
Cl	-2.21400000	-0.47907100	0.00040900
Cl	-0.26544600	2.15868100	-0.01128500
O	0.28691500	-0.25291500	1.99415600
H	-0.05142000	0.54261200	2.42735300
O	0.29075200	-0.28309200	-1.98987900
H	-0.05516900	0.50069200	-2.43798500
H	-2.53328700	0.77887600	0.01511100

1_4

(-1303.899773)

Pt	-0.01419800	0.14532200	0.05068300
N	-0.06817600	-0.66537300	2.00415600
H	0.01718900	0.00175000	2.76792700
H	0.70403200	-1.32952600	2.07610500
H	-0.92897500	-1.19712400	2.13219300
N	-1.57228100	1.54068800	0.28135500
H	-2.45757800	1.08735500	0.50200700
H	-1.65308700	1.93992200	-0.65739900
H	-1.40474700	2.29371400	0.94539100
Cl	1.72494000	-1.28469800	-0.36560300
O	1.45914700	1.65888400	0.38089500
H	1.49914800	1.98689300	-0.54442500
H	2.32547500	1.24601900	0.54676600
O	0.07533600	1.00051000	-1.71772100
H	0.26614800	0.33737000	-2.39745800
Cl	-1.61039800	-1.36847000	-0.49488600

1_5

(-1303.900026)

Pt	0.02395400	-0.15734600	-0.03071600
N	1.77596300	-1.31652800	-0.09364200
H	2.16247300	-1.35118100	0.84910500
H	2.50576700	-0.94123200	-0.69573900
H	1.53847000	-2.26795700	-0.37657500
Cl	-1.95544000	0.97646600	0.14254700
Cl	1.35817400	1.74370600	0.16067200
O	0.05797100	-0.46749200	2.01939600
H	-0.66800500	-1.07849400	2.24136800
H	-0.07994800	0.36459300	2.50528200
N	-0.07862600	0.11382100	-2.01977700
H	0.74662600	0.59575600	-2.37671400
H	-0.21533300	-0.77925000	-2.49436900
O	-0.91141700	-1.91186500	-0.16625800
H	-1.86695300	-1.76564800	-0.23927300
H	-0.89180200	0.70730000	-2.20312200

3_1

(-1456.349553)

Pt	0.51371600	-0.08568300	-0.11662400
Cl	0.14239700	2.13544900	0.38551100

Cl	-0.02496200	-0.75290500	2.01008200
N	1.10387600	0.58914100	-2.02092000
H	2.11953600	0.68504100	-1.96455400
H	0.85792900	0.00874700	-2.81895800
N	0.95187500	-2.09441400	-0.47543500
H	0.62267300	-2.48224500	-1.35616500
H	0.54554800	-2.63323700	0.29039900
H	0.71379000	1.51956900	-2.16824000
O	2.42868500	0.05012100	0.26057800
O	-1.45088500	-0.46174800	-0.75572000
C	-2.54031900	-0.05205500	-0.28510700
O	-2.65992000	0.90088100	0.58125300
C	-3.81853500	-0.68067500	-0.71118300
H	-4.55266300	0.09103400	-0.93436800
H	-3.66019800	-1.32964300	-1.56545700
H	-4.20077700	-1.26490400	0.12829700
H	1.96768100	-2.17736900	-0.41020600
H	2.53806400	0.45127900	1.13634100
H	-1.79797800	1.33102300	0.79784600

3_2

(-1456.339052)

Pt	0.34507800	-0.00000500	0.15555400
Cl	1.13810500	-1.67856900	-1.19897000
Cl	1.13805300	1.67866800	-1.19886600
N	-0.34612300	-1.53006300	1.42505200
H	0.20360400	-1.64227000	2.27412100
H	-1.31814700	-1.31458600	1.66808900
N	-0.34615600	1.52995500	1.42515300
H	-1.31817800	1.31444200	1.66816900
H	0.20356500	1.64210700	2.27423300
H	-0.31159900	2.40861900	0.90775800
H	-0.31155700	-2.40869000	0.90759300
O	2.27992700	-0.00000300	1.02326000
H	2.73567100	-0.77538400	0.64057000
O	-1.37302400	-0.00001300	-0.82960100
C	-2.53547200	-0.00003200	-0.19289100
O	-2.65845800	-0.00005600	1.02844700
C	-3.69591500	0.00004100	-1.14491900
H	-4.30358900	0.88009000	-0.93618500
H	-3.37502300	-0.00057500	-2.18110300
H	-4.30445300	-0.87919700	-0.93532100
H	2.73564600	0.77541800	0.64062100

3_3

(-1456.314815)

Pt	-0.48079200	-0.09434500	-0.13144400
Cl	-0.01730000	-0.76411100	2.00839800
Cl	-0.03495800	2.21361700	0.35182900
N	-1.16431400	-1.96099200	-0.52579300
H	-0.77937100	-2.58622400	0.18229000
N	-0.93977900	0.51466400	-2.09357000

H	-0.20671900	0.25201000	-2.74997900
H	-1.81929900	0.11907700	-2.42144200
H	-1.01783500	1.53212000	-2.09936000
H	-0.79093400	-2.29632200	-1.41276700
O	-2.45822400	0.09242200	0.35620600
H	-2.71449100	-0.85785700	0.25052300
O	1.37967700	-0.52404400	-0.73354700
C	2.47007900	-0.10859200	-0.22921600
O	2.58638800	0.86810300	0.58867400
C	3.72056000	-0.81627500	-0.60390100
H	3.98949300	-1.46423700	0.23390900
H	3.56887500	-1.42472600	-1.48910800
H	4.52496400	-0.09813700	-0.74438500
H	-2.57227200	0.35239100	1.28850300
H	1.72984100	1.37085100	0.73419900

3_4
(-1456.343417)

Pt	0.51059900	-0.06115200	-0.11896900
Cl	0.04899100	2.11893100	0.42567200
Cl	0.12220200	-0.84130400	2.00391900
N	1.00178600	0.70831000	-2.01401600
H	2.01411900	0.84214800	-1.98605000
H	0.74999400	0.15694000	-2.83022600
N	1.06592400	-2.04373300	-0.54822700
H	0.79708000	-2.42308600	-1.45228700
H	0.67856200	-2.63750000	0.18534000
H	0.57266700	1.63020100	-2.09479600
O	2.42069400	0.16558400	0.21048500
O	-1.46282400	-0.48064100	-0.76744500
C	-2.58584900	-0.19884800	-0.29970000
O	-3.64866300	-0.62900800	-0.93779100
C	-2.88650800	0.60890300	0.90461800
H	-3.87058700	0.34858700	1.28343600
H	-2.11839300	0.47899900	1.65814200
H	-2.88935100	1.65858000	0.60461600
H	2.08269200	-2.06161700	-0.45376200
H	2.52323900	0.62361500	1.05914100
H	-3.39053100	-1.12651800	-1.73278800

3_5
(-1456.336950)

Pt	0.47909900	-0.07077500	-0.12958600
Cl	1.06133700	1.89142600	0.89915900
Cl	-0.19794300	-1.01622500	1.84743000
N	1.23619600	0.82816200	-1.87618800
H	2.21056200	0.52514200	-1.91965800
H	0.77733000	0.63013900	-2.76122700
N	0.08733700	-1.95900600	-0.98445600
H	-0.24820700	-1.99985200	-1.94262500
H	-0.58315900	-2.43513900	-0.38346200
H	1.23187700	1.83659900	-1.72124600

O	2.28386500	-0.79833500	0.01508300
O	-1.43736600	0.80072000	-0.40832400
C	-2.55195800	0.29401400	-0.27439500
O	-2.67122800	-1.02378000	-0.31758100
C	-3.76655100	1.12891100	-0.06247700
H	-4.08734100	1.00157900	0.97469100
H	-3.52994600	2.17397800	-0.23095800
H	-4.57931200	0.81115800	-0.71544600
H	0.98263100	-2.44553900	-0.91298100
H	2.60513300	-0.60031100	0.90859600
H	-3.58283700	-1.30621100	-0.14770500

3_6

(-1456.296491)

Pt	0.37476300	-0.15854800	-0.09615400
Cl	1.13051100	2.09015500	-0.57621600
Cl	1.12480600	0.02701500	2.05981000
N	-0.25865100	-0.41304500	-2.08550800
H	0.42396100	-0.99785600	-2.56830400
H	-1.18476700	-0.84595300	-2.08994300
N	-0.21161000	-2.02032600	0.36996500
H	-1.19806000	-2.09998200	0.07852700
H	-0.12297300	-2.14170300	1.38061500
H	-0.32480300	0.46974100	-2.58793400
O	2.05009700	-0.99464500	-0.73925100
O	-1.39244200	0.75604000	0.40511900
C	-2.52562200	0.14984900	0.12326700
O	-2.58692700	-0.94448300	-0.44655800
C	-3.74525100	0.89319100	0.58093500
H	-3.91363700	0.65069700	1.63165900
H	-3.60446400	1.96822100	0.50569100
H	-4.60815300	0.56961000	0.00640700
H	0.41585100	-2.67474500	-0.10070100
H	2.72063400	-0.93976500	-0.04146400
H	0.17578000	2.65666600	0.09349800

4_1

(-916.167167)

Pt	1.07697000	-0.02415900	0.02993000
N	2.50983000	1.41792500	0.40794800
H	2.09518500	2.32830500	0.21473900
H	3.32578800	1.32430300	-0.19398400
H	2.77841400	1.39940600	1.39128400
N	2.30640600	-1.59770900	0.29018700
H	3.04032600	-1.59164900	-0.41840200
H	1.72963800	-2.42975400	0.14324000
H	2.69398800	-1.62937400	1.23220400
O	0.77648300	0.05711100	2.01262600
O	1.69380800	0.03359900	-1.86812000
H	1.11103000	-0.51233700	-2.41401500

H	0.11255600	-0.58399700	2.29324500
O	-0.20095000	1.50870900	-0.30793200
O	-0.25388200	-1.42771900	-0.38051500
C	-1.55046300	-1.21043300	-0.61615100
O	-2.22915000	-2.07734500	-1.09889400
C	-1.44114600	1.34531500	-0.39943600
O	-2.16256500	2.38963700	-0.71281700
C	-2.19740500	0.09958500	-0.12405600
C	-2.54068400	-0.07943100	1.42395500
C	-3.72850300	0.11531700	-0.38770400
C	-3.96979500	-0.48599900	1.01292800
H	-2.49179900	0.87140700	1.95157700
H	-1.93296600	-0.81348100	1.94618400
H	-4.10852800	1.12889100	-0.47040500
H	-4.04161300	-0.47327300	-1.24345200
H	-4.77542900	-0.05499200	1.60015600
H	-4.08549800	-1.56589900	0.97502000
H	-1.59040300	3.16726100	-0.84087500

4_2

(-916.167614)

Pt	-1.07637500	0.01676400	0.03136700
N	-2.35735000	1.61588800	0.32520200
O	-0.64255700	0.22953800	1.97982000
O	0.10813800	-1.53013100	-0.28598500
O	0.31785300	1.37926300	-0.51906600
C	1.53968100	1.10010900	-0.58267200
C	1.43059800	-1.46183000	-0.46338300
O	2.04095100	-2.44606900	-0.78588400
C	2.18353400	-0.15979900	-0.13902300
C	2.52219400	0.00030600	1.41796500
C	3.71783900	-0.21096800	-0.38003900
C	3.97397600	0.34635200	1.03593400
H	-1.86440700	2.46522600	0.05387400
H	-2.60874800	1.70263300	1.30907300
H	-3.18991200	1.55151300	-0.25796600
H	-0.09396500	-0.50350400	2.28727600
H	2.43252800	-0.97714700	1.88987700
H	1.92652800	0.72569800	1.96614700
H	4.01153400	-1.25144000	-0.47867700
H	4.09526700	0.35320400	-1.22717700
H	4.75106300	-0.13984800	1.61870400
H	4.14996400	1.41990500	1.03707400
H	-1.92984900	-2.29638100	0.41614400
N	-2.42830800	-1.40559600	0.48509000
H	-3.17299200	-1.40418600	-0.21285200
H	-2.79877800	-1.30911000	1.42920600
H	-1.26978600	-0.64151200	-2.36769900
O	-1.80048500	-0.03872900	-1.82889900
O	2.34693000	2.03151500	-1.01958600
H	1.84479600	2.83717100	-1.23757500

4_3

(-916.157975)

Pt	-1.07637500	0.01676400	0.03136700
N	-2.35735000	1.61588800	0.32520200
O	-0.64255700	0.22953800	1.97982000
O	0.10813800	-1.53013100	-0.28598500
O	0.31785300	1.37926300	-0.51906600
C	1.53968100	1.10010900	-0.58267200
C	1.43059800	-1.46183000	-0.46338300
O	2.04095100	-2.44606900	-0.78588400
C	2.18353400	-0.15979900	-0.13902300
C	2.52219400	0.00030600	1.41796500
C	3.71783900	-0.21096800	-0.38003900
C	3.97397600	0.34635200	1.03593400
H	-1.86440700	2.46522600	0.05387400
H	-2.60874800	1.70263300	1.30907300
H	-3.18991200	1.55151300	-0.25796600
H	-0.09396500	-0.50350400	2.28727600
H	2.43252800	-0.97714700	1.88987700
H	1.92652800	0.72569800	1.96614700
H	4.01153400	-1.25144000	-0.47867700
H	4.09526700	0.35320400	-1.22717700
H	4.75106300	-0.13984800	1.61870400
H	4.14996400	1.41990500	1.03707400
H	-1.92984900	-2.29638100	0.41614400
N	-2.42830800	-1.40559600	0.48509000
H	-3.17299200	-1.40418600	-0.21285200
H	-2.79877800	-1.30911000	1.42920600
H	-1.26978600	-0.64151200	-2.36769900
O	-1.80048500	-0.03872900	-1.82889900
O	2.34693000	2.03151500	-1.01958600
H	1.84479600	2.83717100	-1.23757500

4_4

(-916.156893)

Pt	1.02387667	-0.06383284	0.03833595
N	2.26169465	-1.17563087	1.30037323
O	0.96234896	1.19041876	1.52726315
O	-0.17402510	1.00075384	-1.12831947
O	-0.39377003	-1.24924440	0.77125722
C	-1.36337333	-1.29749495	-0.17313293
O	-1.39944346	-2.21645401	-0.97058837
C	-1.53934915	0.95711782	-1.13166162
O	-2.12539073	1.75167833	-1.80792975
C	-2.24584793	-0.08130635	-0.25696778
C	-2.74456381	0.47046758	1.11971405
C	-3.71898863	-0.38518679	-0.62221517
C	-4.09108095	-0.25017507	0.87324351
H	1.69156675	-1.96224827	1.61587080
H	2.47123186	-0.59791136	2.11505713
H	3.12565991	-1.54385122	0.90994628
H	0.03054433	1.34630616	1.74497082

H	-2.84796006	1.55431873	1.08360403
H	-2.16226135	0.17857206	1.99164209
H	-4.12494352	0.42019600	-1.22914311
H	-3.89144076	-1.34000113	-1.11151043
H	-4.99294687	0.30753039	1.10924631
H	-4.12937585	-1.21542963	1.37626877
O	1.15012789	-1.51577163	-1.53467414
H	0.36468213	-2.11373718	-1.39708465
H	1.00460290	-1.11725517	-2.40811850
N	2.42129532	1.29084363	-0.69170596
H	2.61272846	1.94057244	0.07298778
H	3.29841148	0.94157530	-1.06982967
H	1.92959277	1.81739149	-1.41777097

4_5
(-916.154851)

Pt	1.09120000	-0.00564800	0.05758200
N	2.53861500	1.46109400	0.29771100
H	2.42652200	2.22570500	-0.36513900
H	3.48717500	1.10132700	0.21127200
H	2.40830100	1.82689900	1.24256300
N	2.40949100	-1.38389800	0.61836300
H	2.50050300	-1.23894900	-1.56095000
H	1.87028500	-2.23291400	0.79642200
H	2.82957600	-1.13511300	1.51289200
O	0.54594600	0.56297000	1.83935600
O	1.81250100	-0.60828600	-1.86136300
H	1.14304900	-1.13935800	-2.32500400
H	0.42572800	-0.22978200	2.38186800
O	-0.26423700	1.43008100	-0.70910600
O	-0.23132300	-1.45261600	-0.17345500
C	-1.53239000	-1.27991700	-0.44838400
O	-2.17572900	-2.21181700	-0.85540500
C	-1.49974600	1.26895300	-0.63416200
O	-2.27706000	2.25910900	-1.00544200
C	-2.21365500	0.06379300	-0.13170400
C	-2.54435000	0.11659500	1.42479400
C	-3.74955700	-0.01376500	-0.37290700
C	-3.95200500	-0.41999200	1.10144800
H	-2.54706700	1.14402500	1.78354800
H	-1.88980800	-0.47908600	2.05247500
H	-4.16498300	0.96622700	-0.58625400
H	-4.05035700	-0.72418200	-1.13564700
H	-4.77812000	0.04515100	1.63162000
H	-4.00913300	-1.49934200	1.21851800
H	-1.73251800	3.02003500	-1.27329600

4_6
(-916.135981)

Pt	1.06003700	-0.03199800	0.01856000
N	2.57275300	0.90774700	1.09119600
H	2.20109200	1.69976400	1.61331600

H	3.31057200	1.25555600	0.47977700
H	2.94715400	0.25749400	1.78133900
N	2.17280400	-1.62810600	-0.50342600
H	3.06990900	-1.40989200	-0.93237600
H	1.59622500	-2.14121100	-1.17653900
H	2.28353400	-2.21012800	0.33064200
O	0.85542300	-1.14113300	1.68339700
O	1.59984000	1.11242200	-1.52107100
H	1.12892300	0.84100500	-2.32214300
H	0.04077100	-0.96367100	2.16549600
O	-0.20637900	1.54213200	0.41441400
O	-0.29638000	-0.96866200	-1.07999500
C	-1.63095900	-0.88882500	-0.96308800
O	-2.33001600	-1.64602200	-1.57618100
C	-1.59116100	1.48885800	-0.14605800
O	-1.98903100	2.49958300	-0.60442400
C	-2.23264700	0.15840100	-0.00687300
C	-2.37076200	-0.44846300	1.44256200
C	-3.78158900	0.21457900	-0.08640400
C	-3.86311000	-0.71727100	1.14141100
H	-2.21152900	0.30410000	2.21417900
H	-1.75538900	-1.32323100	1.63775000
H	-4.14206300	1.22008900	0.11904500
H	-4.20320500	-0.14696900	-1.01827900
H	-4.56455600	-0.43648500	1.92164700
H	-4.03400700	-1.75221500	0.85686700
H	0.18445500	2.31389400	-0.05119000
